# Supplementary material for: Are carbon emissions trading and green financial instruments synergistic? -Comprehensive quantitative research based on content analysis
Source: PLoS One. 2024 Mar 7;19(3):e0298601. doi: 10.1371/journal.pone.0298601 (PMC10919601; doi:10.1371/journal.pone.0298601)
Supplement: S1 Data — (ZIP) [file pone.0298601.s001.zip › Data/地级市Z_F工作报告.zip!/╔┬╬≈/═¡┤¿╩╨/└┤╫╘╣1⁄2╓┌║┼í╛┬φ┐╦╩2╛▌═°í┐_2019.docx]

各位代表：

　　现在，我代表市人民政府向大会报告工作，请予审议，并请市政协委员提出意见。

　　一、2018年工作回顾

　　2018年，是全面贯彻党的十九大精神的开局之年，是铜川发展进程中极不平凡的一年。面对各种风险挑战和繁重发展任务，在市委的坚强领导下，全市上下坚持以习近平新时代中国特色社会主义思想为指导，牢固树立“四个意识”，坚定“四个自信”，坚决做到“两个维护”，紧扣追赶超越和“五个扎实”要求，认真落实“五新”战略任务，统筹推进稳增长、促改革、调结构、惠民生、防风险各项工作，较好地完成了市十六届人大三次会议确定的各项目标任务。生产总值增长6%，地方财政收入增长15.2%，城乡居民人均可支配收入分别增长8.3%和9.2%。

　　（一）转型发展迈出新步伐。加快农业增效、工业转型、服务业高质量发展，落实“土地十条”“金融十条”等配套措施，转型步伐明显加快，经济保持稳定增长。粮食总产28.78万吨，水果产量54.16万吨，全市首单玉米“保险+期货”落地，创建省级畜禽标准化示范场3个，第25届杨凌农高会获奖总量全省第一，印台区荣获全国品牌农业示范区，红土镇被列入全国农业产业强镇示范建设名单。新增规模以上工业企业55户，美鑫30万吨铝镁合金、宜兴达年产150万吨精洗煤等43个工业项目建成投运，煤炭产量稳中有增，规模以上工业增加值增长4.1%。盘活、改造、新建标准化厂房71.27万平方米，国家水泥质量监督检验（铜川）中心建成投运。引进商贸服务业项目75个，荣盛广场等商业综合体建成运营，服务业增加值增长7.4%，社会消费品零售总额增长11.1%。

　　（二）产业集群开创新局面。以规划引领明晰思路、提振信心，加大招商引资，加速项目聚集，签约项目404个，实际引进市域外资金220亿元。140个市级重点项目建成31个，完成投资201亿元，全社会固定资产投资增长13.8%。航天科技、数字经济、高端装备制造等产业集群初具雏形，中国（铜川）商业航天城建设全面启动，12家航天科技企业落户，11个重点企业产业技术研究所挂牌成立，“铜川一号”卫星成功发射，新增高新技术企业4家，高新技术产业增加值增长36.4%，申报发明专利59件，技术合同成交总额2.25亿元，新区获批省级高新技术产业开发区。铜川大数据学院挂牌招生，摩贝、中国安易等18家数字企业全年交易额25亿元。陕西格瑞天成新能源电动车、众晟新能源汽车配件等项目建成投运，达美600万只汽车铝合金轮毂等项目开工。中药材种植面积达到7.7万亩，新丰禾、百年寿等生物医药、健康服务项目建成投运。签约陶瓷项目57个，建成耀瓷大师创意园，成功举办耀州窑创烧1400年纪念活动。完成旅游投资22.3亿元，全市接待游客量、旅游总收入分别增长21%和28%。王石凹煤矿被认定为国家工业遗产，溪山胜境风景区被确定为全国森林康养基地试点建设单位。

　　（三）三大攻坚战取得新成果。扎实推进“双化解”行动，启动了债券发行及资产整合工作，设立首批规模60亿元的市场化股权投资基金。集中力量扶持优势企业挂牌上市，30家企业在陕西股权交易中心交易板挂牌。全市综合债务率下降20%，金融机构不良贷款占比由4.8%下降到1.55%，涉稳风险预警防范率100%。宜君县普惠金融被世界银行确定为全球倡议项目。扎实推进打赢脱贫攻坚战三年行动，成功举办扶贫产品交易会，发展贫困村集体经济组织174个，62个苏陕扶贫协作项目竣工23个，移民搬迁安置群众4905户14780人，“爱心超市”实现贫困村全覆盖，86个贫困村退出，3788户12150人稳定脱贫，宜君县达到脱贫摘帽标准。打响了“青山、蓝天、碧水、净土”四场保卫战，全年优良天数235天，位居关中前列。渭北旱腰带治理成效明显，完成第二次全国污染源普查试点任务，山水林田湖生态保护与修复工程扎实推进，全年植树造林10.8万亩。全面落实河长制，完成城市饮用水源保护区划调整，治理水土流失202平方公里，出境断面水质不断改善。实施重点减排项目44个，单位GDP能耗下降6%，荣膺“2018绿色发展典范城市之首批生态型城市”。

　　（四）城乡面貌发生新变化。加快城市改造提升，南北市区协调发展、新耀一体化迈出新步伐，启动北市区城市“三疏”行动，王益区被列为全省首批城市设计、“城市双修”试点区县。有序推进城市道路“白改黑”，加快打通城区“断头路”，华夏南道、华原西道、开元路建成通车。新增天然气用户13073户，扩大集中供热16万平方米，新增城市绿化31万平方米。川陈公路建成通车，开通旅游公交专线7条、工业园区公交线路2条，改善农村公路380公里，耀州区被命名为“四好农村路”省级示范区。乡村振兴战略深入推进，实施重点项目721个，完成投资9.54亿元，创建最美村1个、示范村21个、精品村35个，王益区被评为“全省改善农村人居环境工作先进区县”，印台区西村荣获“全国生态文化村”称号。

　　（五）改革开放再添新活力。以“放管服”改革为抓手，扎实开展优化提升营商环境“十大行动”，取消项目核准前置条件18项，省市区（县）三级政务服务平台实现互联互通。全年新增各类企业3144户，增长77.6%，44390户市场主体实现“多证合一”。供给侧结构性改革不断深化，设立1亿元困难企业帮扶专项资金，为企业减免税收4.72亿元，13户企业获直供电量9.6亿千瓦时。国资国企改革顺利推进，完成市盐业总公司改制工作，7个国有林场改革通过省级验收，国有企业职工家属区“三供一业”分离移交工作基本完成。农村改革持续深化，土地确权登记办证率达到99.3%。土地供应率连续三年全省第一，节约集约用地走在全国前列。供销合作社综合改革稳步推进。对外开放不断扩大，成立了“一带一路”经贸合作推进办公室及驻海外办事处，在津巴布韦投资建设物流产业园，与日本、韩国、土耳其等国家地区开展了友好交往，首届中国国际进口博览会签约采购金额7.4亿元，全年实际利用外资2016万美元，外贸进出口总额3.01亿元。

　　（六）民生社会事业取得新进展。城镇新增就业10300人，城镇登记失业率3.23%，农村劳动力转移就业66932人次。发放创业担保贷款2.39亿元，扶持1563人实现创业。建成创业孵化基地29个，入驻企业600余家，人才公寓、创业大街建成投用，大学生创业孵化园荣获2018中国优秀“双创”服务平台大奖。普惠性幼儿园占比达到85.2%，义务教育超大班额全面消除，耀州中学、宜君高中通过省级示范高中和标准化高中验收，启动中小学幼儿园规划建设三年行动，新建改扩建中小学校、幼儿园38所，建成投用25所，全市中小学、幼儿园直饮水和寄宿制学校浴室实现全覆盖。成功举办建市60周年系列活动，启动全国文明城市和健康城市创建工作，全民健身馆投入运营，铜川大剧院、博物馆、体育馆、工人文化宫主体封顶，成功创建为国家公共文化服务体系示范区。全市居民健康素养水平达到13.37%，高于全省平均水平2.83个百分点。市人民医院、矿务局中心医院、北京中医药大学孙思邈医院创建为三甲医院，耀州区创建为国家级健康促进示范区，我市连续七届十四年蝉联全国无偿献血先进市。城市、农村低保标准增幅分别为9.8%和15.27%，城镇职工养老、医疗保险参保率99.8%，新农合参保率99%，建成4个城市社区老年人日间照料中心、53个农村幸福院，连续两年入选“中国最具幸福感城市”。

　　（七）自身建设和治理创新取得新成效。坚持把“以案促改”作为重要任务，扎实开展“讲政治、敢担当、改作风”专题教育，切实加强法治政府建设，政府系统风清气正的政治生态得以巩固。深入推进民主决策、依法施政，凡事先调研、再决策，先规划、再落地。全面加强政府效能建设，统一规划权，下放城管权，扎实开展“双百双千”助企帮困活动，各级干部有精神、在状态，抓经济抓发展的氛围日渐浓厚，397件建议提案全部按期办结。坚持阳光行政，大力精文简会，厉行勤俭节约，全市“三公”经费下降9.3%，720名公民代表走进政府，180名大学生到政府机关见习。持续深化城乡基层治理，圆满完成村民委员会换届选举，全面启动“雪亮工程”，扎实推进扫黑除恶专项斗争，全市四级综治维稳中心建成运行，社会治安满意率93.93%，实现“六连升”，王益区荣获“全国法治县（区）创建活动先进单位”。信访突出问题得到有效化解，我市荣获全省2018年度信访工作先进单位，宜君县荣获全国信访工作“三无”县。安全生产实现“双下降”，食品安全救助保险全覆盖。我市被评为“2018全国社会治理创新示范市”。

　　民族、宗教、双拥、审计、人民防空、国防动员、地震、气象、测绘、档案、地方志等方面工作均取得了好成绩新成效。

　　各位代表，2018年的成绩来之不易。这些成绩的取得，是省委省政府坚强领导、关心支持的结果，是市委统揽全局、科学决策的结果，是市人大、市政协及社会各界监督支持的结果，也是全市干部群众精诚团结、共同努力的结果。在此，我代表市人民政府，向全市人民，向各民主党派、各人民团体，向关心支持铜川发展的各界人士，表示衷心的感谢和崇高的敬意！

　　在看到成绩的同时，我们也清醒地认识到，我市发展中还存在不少困难和问题：一是经济发展质量还不高，区县之间、园区之间、产业之间发展不平衡不充分的问题依然存在。二是新旧动能转换不快，传统动能增长减弱，新兴动能释放不足，新经济、新业态尚处于起步阶段。三是社会事业和民生工作还需持续改善，在脱贫攻坚、生态环保、城市改造等方面仍有群众不满意的地方。四是政府自身建设还有待加强，工作质量和效率还有待提升，形式主义、官僚主义还比较突出。我们将采取有力措施，切实加以解决。

　　二、2019年工作思路和预期目标

　　2019年是新中国成立70周年，是决胜全面建成小康社会的关键之年。国家继续实施积极的财政政策和稳健的货币政策，持续推进“一带一路”建设和西部大开发，支持资源型地区经济转型发展，为我们提供了国家层面的战略支撑。省委省政府坚定不移发展“三个经济”，大力推动关中协同创新和西铜融合发展，为我们创造了难得的发展机遇。我市通过规划布局、政策牵引、产业植入和项目带动，产业新型化、集群化态势正在加速形成，民营经济、实体经济、新兴经济的发展活力逐步增强。只要我们抓住重要战略机遇期，扭住发展第一要务不动摇，使一系列规划落子定局，一大批好项目落地生根，一个个产业集群孕育成型，就一定能开创铜川追赶超越、转型发展的新局面。

　　今年政府工作的总体思路是：以习近平新时代中国特色社会主义思想为指导，全面贯彻党的十九大和十九届二中、三中全会及中央经济工作会议精神，按照省委十三届四次、市委十二届五次全会的安排部署，全面落实“五个扎实”和“五新”战略任务，坚持以人民为中心的发展思想，坚持稳中求进工作总基调，坚持新发展理念，坚持深化市场化改革、扩大高水平开放，加快建设航天科技、数字经济、高端装备制造、环保陶瓷、生物医药、文化旅游体育等产业集群，大力实施高新企业速育、民营企业增效、市场主体倍增、固定资产投资、城市人口增容、上市企业飞龙等行动计划，建设交通、物流、教育、医疗、文化区域中心，坚决打好打赢防范化解重大风险、精准脱贫、污染防治“三大攻坚战”，加速实现工业、旅游、城市经济新突破，推动质量变革、效率变革、动力变革，加快经济社会高质量发展。

　　综合考虑各方面因素，今年全市经济社会发展主要预期目标是：生产总值增长7%－7.5%，规上工业增加值增长6.5%－7%，服务业增加值增长9%，农业增加值增长4.5%，全社会固定资产投资增长13%－15%，社会消费品零售总额增长13%，地方财政收入增长12%，城乡居民人均可支配收入分别增长8%和9%，单位生产总值能耗、主要污染物排放完成省上下达的指标任务。

　　做好今年的政府工作，必须解放思想、深化改革、系统思考、综合施策，在三个方面做足文章、下足功夫。

　　一是坚持发展是铜川第一要务的理念不动摇、不松劲，加快培植新动能，加速聚集新优势。培育大企业大集团是推动传统产业改造提质的必由之路，打造产业集群是培育新动能的战略选择，建设区域中心既是提升城市品质之要、发展城市经济之基，更是民之所需、民生实事。这一系列谋划布局，是我们站位全省大局，结合铜川实际，围绕调结构、促转型打出的“组合拳”。全市上下要耐住性子、沉下身子，保持定力、久久为功。要进一步优化发展环境，激发更大干劲，形成强大合力，全市一盘棋，抓出快节奏，干出新成效。要坚持大战略与小规划、大布局与小板块、大愿景与小目标相结合，加快把规划图变为路线图，把路线图变为施工图，推动近期目标出形象、快见效，远期目标早实现、快闭环，通过一年努力，集群化产业布局成规模、见效益，力争进入全省第一方阵，2020年以后大发展、开新局，确保高质量建成“西部传统产业转型升级示范城市”。

　　二是坚持重点突破整体推进的决心不动摇、不松劲，加快实现转型升级，全面提升发展水平。落实“五个扎实”、实现追赶超越就是要在重点领域、重点行业、重点区域求突破上台阶。要力促县域经济争先进位上台阶，耀州区、王益区、印台区年均提升2个以上位次，宜君县提升5个以上位次，新区成为全市产业的领头雁、发展的急先锋。力促园区经济集群发展上台阶，工业园区强身健体，突出引领作用，每个园区新增入园企业10户以上，工业增加值、固定资产投资增速均高于全市10个百分点以上。力促工业经济转型升级上台阶，大企业大集团支撑引领作用明显增强，工业产业集群加速聚集，培育规模以上工业企业60户，产业集群和企业集团对全市经济贡献率提升10个百分点以上。力促旅游经济提质增效上台阶，景区改造提升规划全面落地，文化旅游体育产业集群基本形成全域大闭环、景区小闭环，全市接待游客数量、旅游总收入分别增长15%和20%以上。力促城市经济协同创新上台阶，北市区城市“三疏”行动加快推进，南市区新耀一体化实现新突破，南北市区连接带疏通扩容，物流、电商、医药健康等一批产业园高效运行，让产业有活力，让城市更年轻。

　　三是坚持创新驱动扬长补短的方法路径不动摇、不松劲，加快激发内生动力，全面增强发展活力。加快实施产业振兴“六大行动计划”，是我们补齐发展短板、畅通经济循环、推进高质量发展的“新阵法”。全市上下要不遗余力抓落实，扎实开展“新起点新征程改革开放再出发”经济社会发展实践，瞄准高科技、硬科技招商引资，培育发展科技创新型小巨人企业、瞪羚企业和独角兽企业。精准落实支持民营企业高质量发展的政策措施，全面提升税收治理能力和服务水平，减轻企业税费负担，推动中小微企业茁壮成长。全方位挖掘市场主体增长空间和潜力，力促一批优质企业上市融资，力争上市公司迁址落户或产业导入，加快补齐铜川发展短板，激发转型发展活力。全面推行重点项目市级领导包抓包联机制，力促签约项目快落地、落地项目早开工、开工项目早见效。建立招才引智与招商引资、创业就业协同联动机制，千方百计招人才、增人口、聚人气，以人口聚集带动产业聚集，推动现代金融、科技创新、实体经济、人力资源等协同发展，形成追赶超越的“铜川实践”。

　　三、2019年主要工作任务

　　（一）坚持大集团引领，加快传统产业转型升级。立足传统产业优势，培育大企业大集团，推动煤、电、铝、水泥和食品等传统产业转型升级提质增效。

　　做新清洁煤炭企业集团。支持发展环保生态型煤炭洗选业，700万吨煤炭精洗项目全面投产。加快矿井改造提升步伐，冶坪煤矿基本建成，推动铜川矿业公司等煤炭企业扩链延链、做大做强。全年新增煤炭产能120万吨，原煤产量稳定在2000万吨左右，综合产值达到100亿元以上。

　　做美绿色水泥企业集团。加快水泥企业重组，开工建设金隅冀东、尧柏两条日产万吨水泥熟料生产线等重点项目，声威、凤凰水泥窑协同处置工业固废项目投运，城市垃圾发电项目开工建设。加快生态水泥、环保骨料、装饰混凝土、纤维水泥板材及水泥制品等全产业链融合发展。全年水泥产量2000万吨、骨料600万吨、水泥构件制品100万吨，总产值达到80亿元以上。

　　做大高端铝合金企业集团。依托美鑫公司循环经济产业项目优势，确保30万吨铝镁合金项目投产达效。推动铭帝铝业总部搬迁落地和装饰装修材料生产线提质升档。加快推进天众航汽铝一揽子项目建设，强链扩链，补齐产业链。全年实现产值60亿元以上。

　　做强电力及装备企业集团。支持华能铜川照金煤电公司扩大直供电交易和集中供热范围，力促锦阳电厂满负荷生产，加快推进风电项目和光伏技术领跑基地建设，推动电力产业多元良性发展。大力发展特种电缆、光纤光缆、新能源充电设施等成套装备产业，大唐合力电缆等项目建成达产。全年实现产值35亿元。

　　做亮新型建材企业集团。积极发展新材料产业园区装配式建筑，布局节能环保、轻质高强外墙保温材料等新型建材，加快推动陕建硅酸钙板生产线、天成新型保温材料生产线项目建设，装配式建筑PC生产线、鸥克建材160万平方米抗震墙材生产线建成投用。全年产值突破20亿元。

　　做优食品加工企业集团。鼓励大企业大品牌参与食品行业兼并重组，推动食品加工企业做大做强。发挥旺旺食品龙头带动作用，加快二期、三期项目建设，推动建设台湾旺旺食品产业园。延长苹果、核桃、葡萄等特色地产品深加工产业链，支持桥畔葡萄酒、棋智核桃乳、玉华酒业等扩产提质增效。全年食品企业产值达到20亿元。

　　做大建筑企业集团。加强与陕建集团、西安建工等省内优秀建筑企业合作，支持铜建集团业态创新，扩展产业链，实现“主业专业化、产业多元化”。加快碧桂园、绿地等品牌房企在铜项目建设进度。整合征信良好、质量优质的民营建筑企业资源，建立建筑工程总承包管理平台和资本运作平台，推动全市建筑业上台阶。全年建筑业产值达到50亿元以上。

　　（二）坚持集群化方向，推动高质量发展。以打造产业集群为战术路径，加速培育新动能，再造发展新优势。全年招商签约项目2000亿元，实施重点项目182个，完成投资353亿元，产业类项目投资额占比达到66%。

　　打造航天科技产业集群。围绕卫星制造、卫星测控、卫星组网、终端应用等布局产业链，加速发展航空航天精尖装备与上下游配套产业，推动中国（铜川）商业航天城建设。促进“产学研”深度融合，植入光电集成、人工智能等技术产业，推动建设三级联动科技创新平台，省科技统筹资源铜川分中心、铜川市产业技术研究院建成投用。年内申报发明专利40件，R&D支出占GDP比重达到0.5%，落地航天科技企业20户，新增高新技术企业5家，技术合同成交2亿元以上。航天产业完成投资30亿元。

　　打造数字经济产业集群。加快中国（西部）工业品数字港、铜川数字经济产业园、大数据产业园、京东（铜川）数字经济产业园、电竞小镇、智慧城市等项目建设，新建一批科创平台、孵化中心和试验基地。大力发展“飞地经济”，加快建设“双创”园区，聚集一批数字经济企业，引进电商企业50家以上，落地交易额5亿元以上企业20家。数字经济交易额突破100亿元。

　　打造高端装备制造产业集群。以董家河循环经济产业园、南部产业园、雷家沟老铝厂为主要承载区，拉长高端装备制造产业链，做实“陕西制造、铜川配套”。力促年产1000万片制动摩擦片项目、年产200万件汽车配件制造项目投产运营，百利锂电产业园、万宝集团汽车热加工零部件产业基地、年产50万片盘式制动器项目开工建设。董家河循环经济产业园产值突破100亿元，南部产业园产值实现20亿元。

　　打造环保陶瓷产业集群。以耀州窑文化基地为中心，加快“一带一路”陶瓷产业新城、陈炉陶瓷工业园区、王家河工业园区建设。深化与景德镇陶文旅集团合作，力争东鹏陶瓷等企业入驻铜川。着力在全产业链上做文章，做大做强中高档日用瓷、建筑瓷，年内落地陶瓷企业30户，全力推动“一带一路”陶瓷企业总部基地建设。陶瓷产业产值突破20亿元。

　　打造生物医药产业集群。深入挖掘“药王”文化内涵，积极推进孙思邈研究院建设。扩大黄芩、党参等适生道地中药材种植规模，争取突破10万亩。加快建设国家级中草药植物园、中药材物流园，规划建设药王山中医药产业园、彭镇大健康产业园和新区南部中医药产业聚集区。加快推进天一制药、千禾制药、汉夏生物科技等项目建设，药王针灸产业连锁项目投入运营，引进中医药、生物医药及关联企业30户。生物医药产值突破30亿元。

　　打造文化旅游体育产业集群。加强与知名文化旅游企业集团深度合作，探索实践文化、旅游、体育融合发展路径，创新开发特色旅游产品，加快做强旅游产业。精心实施耀瓷小镇、王石凹工业遗址公园等文化产业项目，支持文化企业发展壮大，加快做优文化产业。策划马拉松、冰雪运动等精品体育赛事，积极推进电子竞技等项目落地建设，加快做大体育产业。新增文化旅游体育类企业50户，文化旅游体育产业综合收入突破150亿元。

　　（三）坚持高点定位，建设宜居宜业新城。着眼发展“三个经济”，加快建设区域交通、物流、教育、医疗、文化五个中心，促进西铜融合发展，打造关中平原城市群新的增长极。

　　建设区域交通中心。加快推进新区青岗岭至惠塬一级公路等重点项目建设，开工建设玉皇阁二号桥、石仁立交等重大交通项目，完善园区景区交通路网，改善农村公路200公里。做好西延高铁、合凤高速铜川段项目服务保障，开工建设342国道印台区崾岘梁至画眉梁二级公路，争取宜君通用机场、小丘货运机场落地建设，加快构建综合立体交通体系。

　　建设区域物流中心。加快建设西北耐物流园、关庄电商物流扶贫产业园、农产品仓储物流中心、陕西果业集团农资产业园，开工建设陕果“一中心两基地”和北市区果品冷链物流项目，力争鼎铭物流园二期年内开工，建成坡头加工贸易产业园。以“互联网+”为导向，设立专项基金，引进知名电商，争创国家电子商务示范基地，打造面向西北的物流产业基地。

　　建设区域教育中心。大力实施教育信息化建设，新建改扩建中小学、幼儿园30所。全力实施高中教育普及攻坚计划，高标准建设王益中学、梅乐园中学。大力发展职业技术教育，建强做优职教中心。争取陕西科技大学、景德镇陶瓷大学在铜设立丝路国际陶瓷学院。鼓励社会力量兴办教育，促进民办教育健康持续发展。加快推进北京外国语大学附属铜川国际学校建设，打造一流教育品牌。

　　建设区域医疗中心。深化医药卫生体制和城市公立医院综合改革，抓好医养结合改革试点，加快推进市中心血站、铜川五官医院、市妇幼保健院迁建项目，市人民医院新区二期建成投运，引进知名医院和重点专科进驻铜川。实施新一轮基层卫生能力提升三年计划，加强镇卫生院、村卫生室和社区医疗机构建设。大力发展智慧医疗、普惠医疗，推广家庭医生签约服务。加强与国家中医药管理局、北京中医药大学等战略合作，积极争取陕西中医药大学在铜建设“一带一路”国际学院。

　　建设区域文化中心。巩固提升国家公共文化服务体系示范区创建成果，加快铜川广播电视技播中心暨公共文化场馆、铜川博物馆建设。高标准规划建设铜川体育场、游泳馆等重点项目，改造提升城市运动公园，启动建设体育专修学院。开工建设市区两级特色文化街区，办好第26届消夏纳凉广场文化活动。加大历史文化遗产保护力度，建立红色文化、耀州窑工艺、中医养生等文化研学基地，提升铜川文化影响力。

　　（四）坚持问题导向，打好三大攻坚战。“三大攻坚战”既是政治任务，也是难啃的“硬骨头”，必须举全市之力攻坚拔寨。

　　打好防范化解重大风险攻坚战。坚持底线思维，增强忧患意识，出台防范化解重大风险攻坚战三年行动计划，打好防范和抵御风险的有准备之战，全力保障经济持续健康发展、社会大局和谐稳定。完善地方金融监管体系，做好重点领域风险防范和处置，持续打击非法集资、金融诈骗等违法活动。完善全市金融风险防控联席会议制度，加强政府性债务风险管控和融资平台监管，加大金融机构不良贷款核销力度，坚决查处各类违法违规举债、担保行为。市本级退出债务风险预警地区名单，全市金融机构不良贷款率维持在1.7%以下。

　　打赢精准脱贫攻坚战。坚持脱贫攻坚思路不变、力度不减，以更实举措巩固脱贫成果，决战决胜脱贫攻坚。对标“两不愁、三保障”，突出产业扶贫根本，大力发展果业、畜牧业、设施大棚等扶贫主导产业，夯实贫困人口稳定脱贫基础。大力发展电商扶贫，力争农产品网络销售额达到2亿元，带动贫困群众1000户以上。加大7个深度贫困村和特殊困难群体脱贫攻坚力度，着力激发贫困人口内生动力。创建8个就业扶贫基地，带动贫困劳动力实现就业1000人以上。深化盐铜扶贫协作，推动帮扶项目取得实效。完成2029户5606人脱贫、23个贫困村退出任务，印台区、耀州区达到脱贫摘帽标准，王益区、宜君县巩固提升脱贫成果，全市实现整体脱贫。

　　打好污染防治攻坚战。深刻汲取秦岭北麓违建别墅教训，继续抓好中省环保督察反馈问题整改，系统推进减煤、控车、抑尘、治源、禁燃和增绿行动，持续抓好山水林田湖生态保护与修复，加快流域治理和管网改造，实施一批重点污水治理工程，加大漆水河川口至耀州城区段治理力度，积极推进新区水资源综合利用项目，确保出境断面水质达标。加快气化铜川建设，煤改电、煤改气走在全省前列。加强土壤污染防治，加快生态红线划定工作。抓好国家森林城市、国家园林城市创建，全年植树造林10万亩以上，治理水土流失150平方公里。

　　（五）坚持系统思维，加快乡村全面振兴。聚焦关键，明确方向，统筹推进产业振兴、人才振兴、文化振兴、生态振兴、组织振兴，加快打造产业兴旺、生态宜居、乡风文明、治理有效、生活富裕的新农村。

　　大力发展特色现代农业。粮食播种面积稳定在86万亩以上，总产量达到20万吨以上。培育打造绿色生态农产品生产基地，加快9个生态循环农业示范区、20个绿色有机农产品生产基地建设。大力发展生态高效畜牧业、干杂果、食用菌等区域特色产业，建设优质高效设施农业生产基地，支持印台区创建国家现代农业产业园区。组建国有农业投资公司，建设“一带一路”果品深加工产业园、果用物资产业园。加强与红星美羚、新希望等集团公司合作，壮大提升肉羊、蛋鸡、生猪等产业联盟。扩大铜川果业宣传，打造“铜川苹果”“铜川大樱桃”“宜君核桃”“耀州花椒”等区域公共品牌，做大做强优质果品基地，不断提高农业产业发展水平。

　　全面深化农村改革。深化农村土地“三权分置”改革，妥善解决农村土地确权登记颁证工作遗留问题,保障所有权，稳定承包权，放活经营权。深化“三变”改革试点工作，深入推进农村集体产权制度改革，支持农村集体经济发展壮大，全市50%以上的村实现经营性收入稳定增长。加快农业经营体系改革，积极培育新型经营主体，完成100个家庭农场、农民专业合作社、农业产业化龙头企业的培育和规范提升任务。积极推进农村配套改革，大力实施“3+X”工程，完善推进农业适度规模经营配套措施，为乡村振兴提供政策保障。创新乡村人才工作体制机制，培育一批带动能力强的“土专家”“田秀才”，发展壮大乡村人才队伍，引领乡村振兴战略高质量落地。

　　建设生态宜居乡村。学习借鉴浙江“千村示范、万村整治”工程先进经验，健全自治、法治、德治相结合的乡村治理体系，深入推进农村人居环境整治，实施垃圾污水处理、厕所革命、村容村貌提升等重点工程，加快农村基础设施和公共服务配套建设。挖掘乡村历史文化资源，优化文化服务体系，推动乡村文化振兴。加快打造照金、小丘、关庄、太安、陈炉、王石凹、黄堡等10个产城融合特色小镇，建设100个美丽乡村、20个乡村振兴战略示范点。

　　（六）坚持区域协调，提升城市品位。以城市规划为引领，以巩固提升创国卫成果为重点，以完善城市功能为目标，在新型城镇化、布局新经济上求实效，全面提升城市建设管理水平，着力打造产城融合、城乡互融、南北交融的现代化城市。

　　提质扩容南市区。以互联互通、基础设施共享为目标，推进新耀一体化进程，加快打造全市政治、经济、文化中心。大力实施城中村改造项目，加快城市提质工程，推进海绵城市和智慧城市建设，完善城市管理信息系统，开工建设西环公园，铜川书画公园建成开放，美佳华商业广场、悦豪五星级酒店、铜川大厦建成营运。加快实施耀州区“工业强区、果业富民、文化兴耀、城镇化带动”战略，推进耀州古城改造，启动“绿染耀州”行动，培育现代物流、医药保健、文化旅游等新兴产业。推动新区创建国家级经济技术开发区，新区人口达到20万人，打造改革的主战场、开放的新高地。

　　改造提升北市区。紧抓国家支持老工业区搬迁改造机遇，加快实施地下综合管廊建设、棚户区改造等重点工程，扎实推进老旧小区、小街巷人性化整治和夜景提升、景观绿化工程，实施漆水河两岸拆改项目，加大城市供水、供热、供气、污水和垃圾处理等基础设施建设力度，强化公共服务，优化功能配套。抓好王益区“城市双修”，高质量打造黄堡百亿产业集群。加快聚集印台区城市人气，盘活老矿山老厂房，规划建设铸锻造产业园区。支持王益区印台区协同打造百亿“城市商圈”，力推城市经济突破发展。

　　加快打造新宜君。高质量修编县城总体规划，实施好高铁引线、210国道宜君过境公路等重点项目，推动“秀美山城”建设再上新台阶。扎实推动现代农业、避暑康养、新兴能源、中医药种植加工等基地建设。大力发展民宿经济，提升哭泉旱作梯田景区建设水平，创建一批旅游示范村、特色标准化星级农家乐，建成一批集乡村振兴、民宿旅游为一体的康养示范村，全力打造“避暑康养名城”，加快建设县域经济绿色转型发展先行区、幸福产业发展示范区。

　　（七）坚持产城融合，发展全域旅游。以创建国家全域旅游示范市、国家中医药健康旅游示范市为契机，加快实现旅游发展全域化、旅游供给品质化、旅游治理规范化。

　　建设高品质景区。大力实施核心景区上档升级工程，力争照金香山景区进入国家5A级景区创建名录。依托优势资源，突出主题特色，打造避暑度假、文化体验、运动探险和城市休闲等多样化旅游业态。加快药王山养生体验中心、陈炉古镇景区游客服务中心等项目建设，力争照金旅游观光火车等项目开工建设。加速打造金锁石林、花溪谷、孔雀谷、马咀等特色景区，溪山胜境风景区、孟姜塬秦人村落建成开放。完成重点旅游项目投资20亿元以上。

　　提升高质量服务。围绕“吃、住、行、游、购、娱”等旅游消费需求，全面提升旅游基础设施和公共服务质量。完善智慧旅游、智慧城市服务体系，建成智慧旅游信息化平台。加快实施旅游名小吃品牌创建工程，推进特色美食商业街区建设。规划建设精品民宿、温泉酒店。加快旅游交通集散中心、旅游轨道交通、全域旅游环线、景区停车场等配套服务设施建设。加强旅游人才队伍建设，提升旅游管理服务水平。支持大型文化旅游企业在铜扩大投资，建设全省旅游重要目的地。

　　做活多维度营销。创新旅游宣传方式，提高铜川旅游知名度和感召力。挖掘红色照金、药王养生、耀瓷文化等深厚底蕴，丰富全域旅游元素，开发铜川旅游产品，包装推广精品旅游项目与线路，打造红色教育、踏青赏花、避暑休闲、金秋旅游、冬日冰雪等旅游品牌，推动研学、游学、康养、避暑等旅游活动齐头并进。举办第五届中国孙思邈中医药文化节、“一带一路”耀州窑国际陶瓷艺术周、“照金精神”全国巡展等系列活动，提升药王山庙会、玉华宫冰雪节等特色活动水平，办好会展经济，带活全域旅游。

　　（八）坚持深化改革，全面扩大开放。用好改革开放“关键一招”，冲破思维观念围墙，打开解放思想“闸门”,加快释放发展活力。

　　打造一流营商环境。全面实施优化提升营商环境三年行动计划，持续推进“十大行动”，敢于破旧、善于立新，打破“旋转门”“弹簧门”，破除生产要素自由流动障碍。积极推进一体化在线政务服务平台建设，全面提升政务服务规范化、便利化水平。深化“放管服”改革，坚持“一网通办”“最多跑一次”。加强“双随机、一公开”联合监管，健全大市场综合监管工作格局，打造审批事项最少、工作流程最优、办事效率最高、服务质量最好的营商环境，让企业家真切感受到温度和力度，让更多的人看好铜川、投资铜川。完成“智慧金融·普惠铜川”项目，用好风险补偿基金，提升信贷服务水平，持续增强金融服务实体经济的能力。建立第二批股权投资基金，规模达到100亿元，在新三板、区域性股权市场交易板挂牌企业达到40家以上。全年新增市场主体3万户以上，新增私营企业1000户以上，培育“五上企业”200户，民间投资增长20%以上，非公经济占比达到50%。

　　推动重点领域市场化改革。注重改革系统性、整体性、协同性，大幅减少政府对资源的直接配置，充分发挥市场在资源配置中的决定性作用。加强社会信用体系建设，构建信用承诺、信息公示、分级分类、联合奖惩的信用监管机制。按照“完善治理、强化激励、突出主业、提高效率”的国企改革方针，“一企一策”激发企业发展活力。深化国资改革，探索改组国有资本投资、运营公司，完善国资监管机制。采取股权转让、增资扩股、出资新设等多种方式，积极稳妥推进混合所有制改革。加快金融、科技、教育、文化、医疗等体制机制改革，抓好产权制度保护改革试点工作。加大闲置土地处置力度，提高土地使用效益。

　　打开对外开放新局面。坚持开放办市理念，借力陕西自贸试验区建设，发挥丝绸之路经济带节点城市优势，加强与“一带一路”沿线国家和地区交流合作，构建多元化对外开放新高地，打造高水平对外合作新平台，推动外向型经济高质量发展。鼓励存量企业、现有资本市场引进国外投资，加快形成大型外贸企业集团。扩大苹果、陶瓷、中药材等“铜川产品”出口规模，推进煤炭、电力等企业域外转移,在越南建成水泥营销网络。与美国约巴琳达市、韩国奉化郡开展友好合作，与日本、土耳其等国家地市缔结友好城市，加强与国际友城的人文交流和经贸合作，办好日本大阪经贸活动周。全年实际引进市域外资金260亿元，实际利用外资1亿美元以上，进出口贸易总额增长10%以上。

　　（九）坚持改善民生，持续增进群众福祉。积极回应群众关切，办好为民利民实事，让铜川人民的获得感、幸福感、安全感与日俱增。

　　积极推进稳就业促增收。把稳就业摆在突出位置，扩大政策性金融支持，促进多渠道就业。统筹做好高校毕业生、退役军人、农村转移人员、城镇困难人员和残疾人的就业安置、服务管理和权益保障工作。巩固省级创业型城市创建工作，加快建立科技成果众包、众筹平台，新建一批专业化众创空间，提升创业带动就业能力，着力打造“双创”升级版。多渠道增加城乡居民收入，完善城乡居民增收综合配套政策，拓宽财产性收入渠道。城镇新增就业1万人，城镇登记失业率控制在4%以内。

　　扎实抓好健康城市建设。建立健全健康城市建设工作推进机制，持续开展食品放心行动、全民健身行动、全民控烟行动、保卫蓝天行动、合理膳食行动和城乡环境卫生整治行动，积极建设健康步道、主题公园、健康小屋，加快实施大唐养生小镇、市老年服务中心、照金锦园颐舍等项目，启动中国孙思邈老年健康公园建设，争取荣华康养社区项目落地。提升全民预防保健水平，积极推进“健康细胞”建设，推动人的健康与城市建设协调发展。

　　不断完善社会保障体系。大力实施全民参保计划，持续推进机关事业单位养老保险制度改革。统筹抓好扶老、助残、救孤、济困、优抚等工作，推动慈善事业健康发展，建设6个省级标准化示范社区、9个智慧社区、6个农村社区服务中心、4个残疾儿童康复救助机构。加快农村幸福院改造提升，推进县级示范性养老机构建设。健全农村留守儿童、妇女和老年人关爱服务体系。实施市区两级殡仪馆及公益性公墓项目。扎实推进保障性安居工程建设和分配管理，积极开展住房租赁试点，发放城镇住房保障、租赁补贴4000户。

　　加快创新社会治理格局。扎实推进全国文明城市创建工作，实施城市精细化管理、文明交通、文明旅游、诚信建设等十大行动，抓好新时代文明实践中心试点建设，让群众共建共享美好新生活。深化平安铜川建设，深入开展扫黑除恶专项斗争。持续推进法治铜川建设，抓好“七五”普法工作。全面提升信访基础业务水平，积极预防化解社会矛盾风险。加强社区治理创新，优化提升社区网格化管理。以提高劳动者和公民的安全素质为基础，强化安全生产责任体系、公共安全体系、双重预防体系建设，提升应急救援和防灾减灾救灾能力，坚决遏制重特大安全事故。加强食品药品安全监管，保障群众饮食用药安全。扎实做好双拥、人民防空等工作，推动军民融合发展。

　　四、全面加强政府自身建设

　　落实全面从严治党要求，深入推进政府职能转变，讲政治、敢担当、改作风，全面提升政府工作质量和水平。

　　加强政治建设。树牢“四个意识”，坚定“四个自信”，坚决做到“两个维护”，认真贯彻习近平总书记的重要指示批示和党中央的决策部署，精准落实省市委各项工作任务和要求，把党的领导贯彻到政府工作的各方面、各环节。深入开展“不忘初心、牢记使命”主题教育，严肃党内政治生活，严明政治纪律，严守政治规矩，强化政治责任和政治担当。

　　加强效能建设。抓实抓好理论学习，提高战略思维、辩证思维、创新思维，努力建设学习型政府。以市级机构改革为契机，进一步优化机构设置和职能配置，理顺各级各部门职责关系。扎实推进行政职能事业单位改革、相对集中行政许可权改革、综合行政执法体制改革，完成机构改革各项任务。加快政府职能转变，坚持高质量、高标准抓落实，学会在复杂局面中、监督约束下干工作，构建职责明晰、协同高效的政府治理体系，加快建设现代化服务型政府。

　　加强法治建设。强化法治思维，深化依法行政，完善政府立法体制，严格执行重大行政决策法定程序。认真履行行政复议职责，严格、规范、公正执法，做到新官理好旧账，全面维护政府公信力。自觉接受人大依法监督和政协民主监督，主动接受群众和舆论监督，深入听取人大代表、政协委员意见建议，认真办理建议提案，始终与人民群众想在一起、干在一起。

　　加强作风建设。认真落实中央八项规定精神和省市委实施办法，大兴求真务实之风，增强调查研究实效，压减考核频次，为基层减压松绑。畅通群众与政府互动渠道，办好政策例行吹风会。抓好廉政建设，履行“一岗双责”，加大经济责任审计力度，健全厉行节约反对浪费长效机制，“三公”经费只减不增。用好“三项机制”，强化监督执纪问责，持续推动作风转变，确保干部清正、政府清廉、政治清明。新的一年，一定要让认真干事、高效干事、干净干事在铜川大地蔚然成风。

　　各位代表！责任呼唤担当，使命引领未来。让我们更加紧密的团结在以习近平同志为核心的党中央周围，在市委的坚强领导下，不忘初心、牢记使命，保持韧劲、担当实干，奋力开创铜川追赶超越转型发展的新局面，以优异成绩迎接中华人民共和国成立70周年！
